# US*Bombus*, a database of contemporary survey data for North American Bumble Bees (Hymenoptera, Apidae, *Bombus*) distributed in the United States

**DOI:** 10.3897/BDJ.3.e6833

**Published:** 2015-12-30

**Authors:** Jonathan B. Koch, Jeffrey Lozier, James P. Strange, Harold Ikerd, Terry Griswold, Nils Cordes, Leellen Solter, Isaac Stewart, Sydney A. Cameron

**Affiliations:** ‡ Utah State University, Logan, United States of America; § USDA-ARS, Logan, United States of America; | University of Alabama, Tuscaloosa, United States of America; ¶ University of Bielefeld, Bielefeld, Germany; # University of Illinois, Illinois Natural History Survey Prairie Research Institute, Urbana, United States of America; ¤ Black Hawk College, Galva, United States of America; « University of Illinois, Urbana, United States of America

**Keywords:** Anthophila, Apoidea, bees, native, standardized survey, North America, Nearctic, pollinators

## Abstract

**Background:**

Bumble bees (Hymenoptera: Apidae, *Bombus*) are pollinators of wild and economically important flowering plants. However, at least four bumble bee species have declined significantly in population abundance and geographic range relative to historic estimates, and one species is possibly extinct. While a wealth of historic data is now available for many of the North American species found to be in decline in online databases, systematic survey data of stable species is still not publically available. The availability of contemporary survey data is critically important for the future monitoring of wild bumble bee populations. Without such data, the ability to ascertain the conservation status of bumble bees in the United States will remain challenging.

**New information:**

This paper describes US*Bombus*, a large database that represents the outcomes of one of the largest standardized surveys of bumble bee pollinators (Hymenoptera, Apidae, *Bombus*) globally. The motivation to collect live bumble bees across the United States was to examine the decline and conservation status of *Bombus affinis*, *B. occidentalis*, *B. pensylvanicus*, and *B. terricola*. Prior to our national survey of bumble bees in the United States from 2007 to 2010, there have only been regional accounts of bumble bee abundance and richness. In addition to surveying declining bumble bees, we also collected and documented a diversity of co-occuring bumble bees. However we have not yet completely reported their distribution and diversity onto a public online platform. Now, for the first time, we report the geographic distribution of bumble bees reported to be in decline (Cameron et al. 2011), as well as bumble bees that appeared to be stable on a large geographic scale in the United States (not in decline). In this database we report a total of 17,930 adult occurrence records across 397 locations and 39 species of *Bombus* detected in our national survey. We summarize their abundance and distribution across the United States and association to different ecoregions. The geospatial coverage of the dataset extends across 41 of the 50 US states, and from 0 to 3500 m a.s.l. Authors and respective field crews spent a total of 512 hours surveying bumble bees from 2007 to 2010. The dataset was developed using SQL server 2008 r2. For each specimen, the following information is generally provided: species, name, sex, caste, temporal and geospatial details, Cartesian coordinates, data collector(s), and when available, host plants. This database has already proven useful for a variety of studies on bumble bee ecology and conservation. However it is not publicly available. Considering the value of pollinators in agriculture and wild ecosystems, this large database of bumble bees will likely prove useful for investigations of the effects of anthropogenic activities on pollinator community composition and conservation status.

## General description

### Purpose

The purpose of this database is to make available data associated with bees of the genus *Bombus* in the United States. The dataset was developed during a nationwide assessment of bumble bee health and conservation status (Cameron et al. 2011). The dataset represents a systematic survey that promises to be useful in future investigations of bumble bee ecology, conservation and policy.

## Project description

### Title

US*Bombus*, a database of contemporary survey data for North American Bumble Bees (Hymenoptera, Apidae, *Bombus*) distributed in the United States

### Personnel

Jonathan Koch (author), Jeffrey Lozier (author), James Strange (author), Harold Ikerd (database manager, author), Terry Griswold (author), Nils Cordes (author), Leellen Solter (author), Isaac Stewart (author), Sydney Cameron (author).

### Study area description

**Figure 1.**
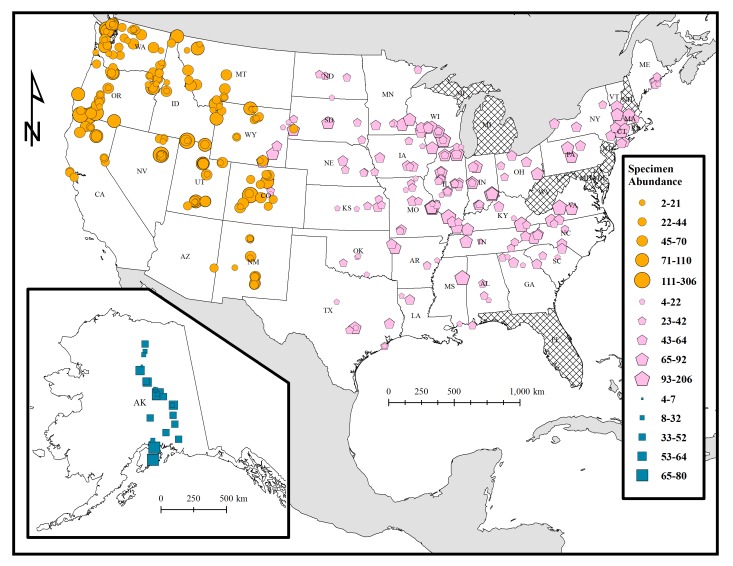
Distribution of bumble bee surveys in the contiguous United States and Alaska. Size of symbol represents the abundance of bumble bees detected. US states not included in the dataset are cross-hatched.

**Figure 2.**
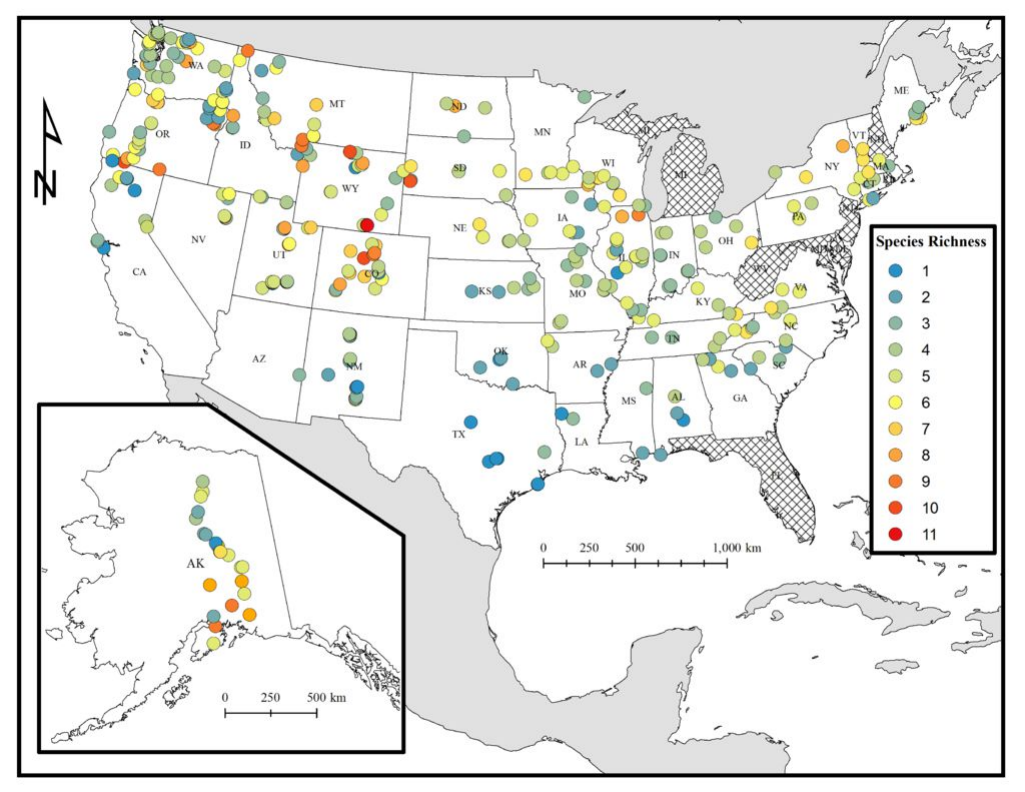
Distribution of bumble bee species richness detected in surveys in the contiguous United States and Alaska. Warmer colors represent high species richness whereas cooler colors represent low richness. Species richness is simply defined as the number of different species detected at a study site. US states not included in the dataset are cross-hatched.

**Figure 3.**
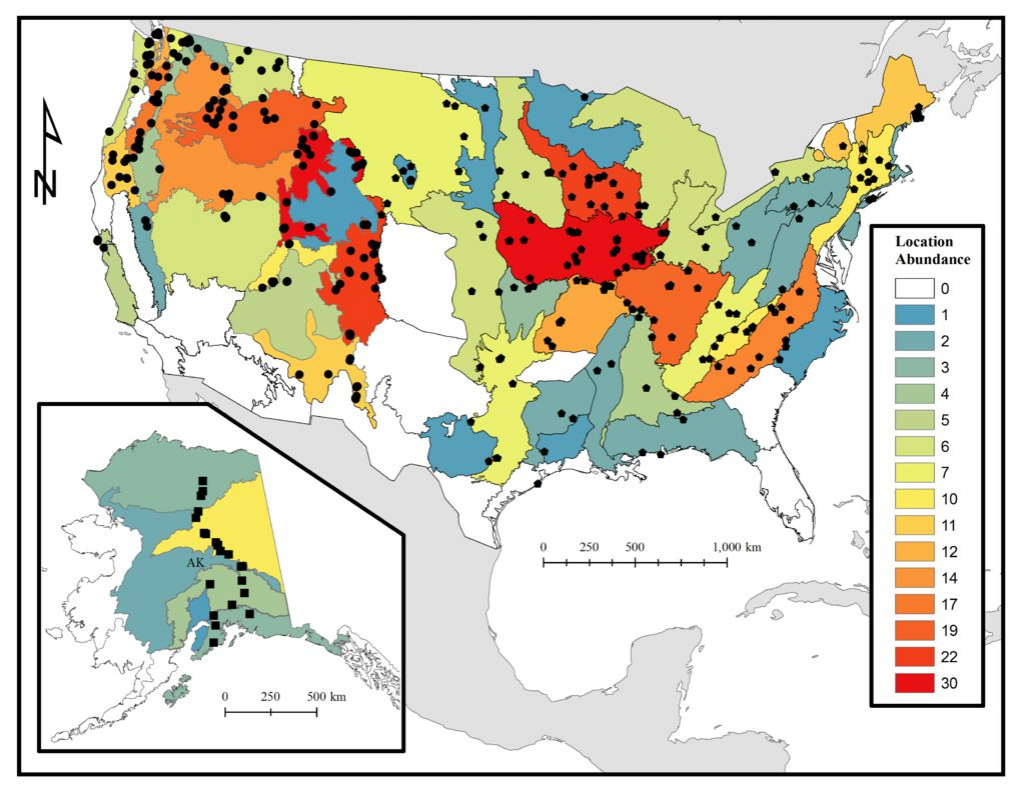
Survey site abundance per World Wildlife Fund ecoregion (Olson and Dinerstein 2002, Olson et al. 2001). Black hexagons = eastern survey, black circles = western survey, black squares = Alaska survey.

This dataset covers a wide range of ecoregions found throughout the continental United States and Alaska, from 29° to 68° latitude and -150° to -68° longitude (Figs 1, 2). Bumble bees reported in this dataset were surveyed in wild, urban, and agricultural landscapes across 41 states from 2007 to 2010. A special effort was made to document bumble bees distributed in US national parks and other federally protected areas, as these lands would likely have been less impacted by anthropogenic land-use change, agricultural intensification, and zoonotic diseases transmitted from commercially reared bumble bees. Nine states and Washington D.C. are not represented in our systematic survey primarily because they were relatively close to states and ecoregions that were intensively sampled (Figs 1, 3). The states not included in this survey and database are Delaware, Florida, Maryland, Michigan, New Hampshire, New Jersey, West Virginia, Rhode Island. Hawaii was not surveyed as bumble bees are not found on this archipelago.

We describe the distribution of bumble bees based on political boundaries and ecoregions that have been developed by the World Wildlife Fund for Nature (WWF) (Olson et al. 2001). A total of 55 ecoregions were surveyed in our national study of bumble bees (Fig. 3). In our survey *B. sandersoni* was detected only in the Appalachian-Blue Ridge Forests ecoregion, and is represented by a single specimen. However, it is likely that we did not survey at an optimal time for *B. sandersoni* as it has been recorded to be in high abundance in some parts of New York, New England, Tennessee, and North Carolina (Hatfield et al. 2015). Furthermore, given that multiple eastern North American bumble bees converge on similar color banding patterns, it is possible that we may have misidentified them in the field (Williams et al. 2014​). In contrast, *B. griseocollis* was detected in 29 ecoregions across the conterminous United States, representing the species with the most ecoregion-diverse distribution in this dataset (Table 1). The initial goal of our study was not to survey across all North American ecoregions equally, but rather investigate ecoregions and states where historic abundances of suspected declining North American bumble bee species were high (Cameron et al. 2011). Based on WWF ecoregions, 62% and 18% of the bumble bees surveyed were collected in critically endangered and vulnerable ecoregions in the United States, respectively (Table 1) (Olson et al. 2001). Only 20% of the surveyed bumble bees were distributed in habitat that has been identified by the WWF as ecoregions that are relatively stable or intact (Table 1). In the western United States, most surveys took place in alpine environments (*e.g.*, Cascade, Sierra-Nevada, and Rocky Mountains) and high elevation basins and plateaus (> 500 m). In the eastern United States, surveys were conducted across a variety of different habitats including prairies and deciduous forests. In Alaska, bumble bees were primarily surveyed in the tundra and taiga, specifically adjacent to large rivers (Fig. 3​) (Koch and Strange 2012).

### Design description

The purpose of the dataset is to make available data associated with a standardized survey of bees of the genus *Bombus* in the United States. That database was developed during the course of an assessment on the conservation status, disease ecology, genetic diversity, and decline of the following North American bumble bees: *B. affinis*, *B. occidentalis*, *B. pensylvanicus* and *B. terricola* (Cameron et al. 2011, Lozier et al. 2011, Cordes et al. 2012, Koch and Strange 2012). The authors Jonathan Koch, James Strange, Terry Griswold, and their field crew primarily collected bumble bees in the western U.S.A. and Alaska while Sydney Cameron, Jeffrey Lozier, Nils Cordes, Leellen Solter, Isaac Stewart and their field crew collected bumble bees in the eastern U.S.A. (Fig. 1). Bumble bees collected by the western group were identified, labelled, pinned, and curated into the US National Pollinating Insect Collection housed at the USDA-ARS Pollinating Insects- Biology, Management, and Systematics Research Laboratory (PIBMSRL) in Logan, Utah. Bumble bees collected by the eastern group were identified in the field to species and released after the survey was completed. Specimens were retained in the western United States and Alaska surveys as several species are cryptic and notorious for misidentification (Koch and Strange 2012). In the eastern United States survey, bumble bees were only retained if the specimens could not be identified to species with complete confidence. Eastern specimens were released as bumble bees could be confidently identified to species using field guides and taxonomic keys. Specifically, specimens of imperiled bumble bees identified in the Cameron et al. (2011) study, as well as *B. vosnesenskii*, *B. bifarius*, *B. bimaculatus*, and *B. impatiens* were retained for population genetic analysis and pathogen surveys. Species identifications were made by the authors with taxonomic keys (Stephen 1957, Thorp et al. 1983, LaBerge and Webb 1962, Mitchell 1962, Medler and Carney 1963, Chandler and McCoy 1965, Husband et al. 1980, Williams et al. 2008, Williams et al. 2014).

Specimen data in the US*Bombus* dataset has been digitized and entered into the US National Pollinating Insects Database (USNPID). Bumble bees collected by the western group have been affixed a six digit matrix barcode with the acronym BBSL. The acronym BBSL (Bee Biology and Systematics Laboratory) is in reference to a previous title of the PIBMSRU. Each physical specimen and associated data is represented by a single BBSL barcode. Bumble bee occurrence and abundance data collected by the eastern group have been incorporated into US*Bombus* dataset in a manner different than the bumble bee specimens collected by the western group. For the eastern data each unique barcode represents the combination of one species with a single collection event (*i.e*., specific field site and date) with the abundance of each sex (male or female), and caste (queen or non-queen) recorded. These survey events have a six digit matrix barcode with the prefix EBOD (Eastern *Bombus*). Both eastern (EBOD) and western (BBSL) specimen data have been entered into the USNPID using data entry forms with Microsoft Access 2008 r2. The USNPID represents one of the largest digital repositories of pollinating insects globally and has been used in numerous ecological, agricultural, and taxonomic investigations (*e.g.*, Griswold et al. 2014).

All locations were georeferenced with a Garmin GPS unit in the field with the coordinate form of decimal latitude and longitude in the WGS84 datum. In this paper specimen records are represented geospatially using ArcGIS and WWF Biotic Regions (Figs 1, 2, 3) (Olson et al. 2001). The data is reported in Darwin Core (DWC) format on the Pensoft IPT Data Hosting Center, http://ipt.pensoft.net/ipt/resource.do?r=usbombus.

### Funding

United States Department of Agriculture grant CSREES-NRI 2007-02274.

## Sampling methods

### Study extent

This dataset was primarily developed to determine the extent of bumble bee decline in the United States. Thus, we did not survey in areas that have historically been under-sampled for bumble bees, nor did we survey well-sampled areas outside of the known ranges of the four focal species suspected to be in decline (Cameron et al. 2011. Much of our survey efforts were guided by natural history specimen data that was digitized retroactively (Grixti et al. 2009, Koch and Strange 2009, Koch 2011). The intent to survey in areas that were once populated with currently rare and declining bumble bee species was to determine changes in genetic structure over time, disease ecology, and population abundances (Lozier and Cameron 2009, Cordes et al. 2012, Cameron et al. 2007). Thus we sampled across both latitude and elevation gradients in a way that maximized our ability to detect and capture bumble bees when colony growth was at its maximum in the summer months of the northern hemisphere.

### Sampling description

Specimens represented in the US*Bombus* dataset are the result of systematic surveys conducted by researchers at the USDA-ARS-PIBMSRL, Utah State University, University of Illinois, and Illinois Natural History Survey. Surveys were conducted primarily using sweep nets to capture bumble bees on flowers and in flight. All surveys were timed and conducted for at least 0.5 hours (average of ∼1 ± 0.5 SD survey hours per site). Surveys were conducted by walking through floral patches and collecting all observed bumble bees without consideration of species identity. Site selection was based on locality data present on natural history collections and species distribution models. Specimens were collected with aerial nets while in flight or while foraging at flowers; then, they were placed in vials and chilled on ice until the end of the collection period. This dataset represents a total of 512 collector hours. Survey methods are further described in Cameron et al. 2011). The number and name of surveyors can be queried from the USNPID by contacting the database manager associated with this data publication.

### Quality control

All unrecognizable individuals collected in the field were carefully examined by the authors using taxonomic keys and field guides (Husband et al. 1980, LaBerge and Webb 1962, Mitchell 1962, Medler and Carney 1963, Thorp et al. 1983, Williams et al. 2008, Chandler and McCoy 1965, Stephen 1957). The authors are considered to be authorities in bumble bee identification in North America (Koch et al. 2012) and globally (Williams et al. 2008).

### Step description

All specimens described in this dataset have been batch entered into the USNPID following the flowchart in Fig. 6​. With the exception of data collected by the eastern group (University of Illinois and Illinois Natural History Survey), specimen identification and subsequent update to the database occurred after record and event metadata had been entered into the USNPID. Bumble bee identification and associated metadata of bumble bees collected by the eastern group were retroactively captured from a spreadsheet and imported in the USNPID. In the USNPID dataset bumble bee queens are denoted by the Q identifier (0 = False, -1 = True). Workers and Queens are denoted by the F identifier as a quantity (0 - ∞) and males are denoted by the M identifier as a quality (0 - ∞). Values greater than one in these fields (M, F) indicate the total abundance of the specimens associated with that caste in the survey event and is specific to occurrence records associated with the EBOD prefix. Quantities were mapped to the Darwin Core DWC field "Individual Count" with cast and sex mapped to the DWC field "Sex" (Female, Female Queen, Male, Unknown Sex).

## Geographic coverage

### Description

This dataset includes occurrence records of bees in the genus *Bombus* across 41 states in the contiguous United States and Alaska. Surveys have taken place over a wide elevation gradient, starting at near-sea level sites including Galveston, Texas and San Juan Islands, Washington to 3500 m a.s.l. in Gothic, Colorado. Considerable effort was also made to survey multiple bumble bee communities north of the Arctic Circle (68° latitude) in Alaska. However, the majority of the field sites represented in this dataset are found throughout in grassland and alpine biomes of the contiguous United States (Figs 1, 2, 3).

### Coordinates

68° and 29° Latitude; -68° and 150° Longitude.

## Taxonomic coverage

### Description

**Figure 4.**
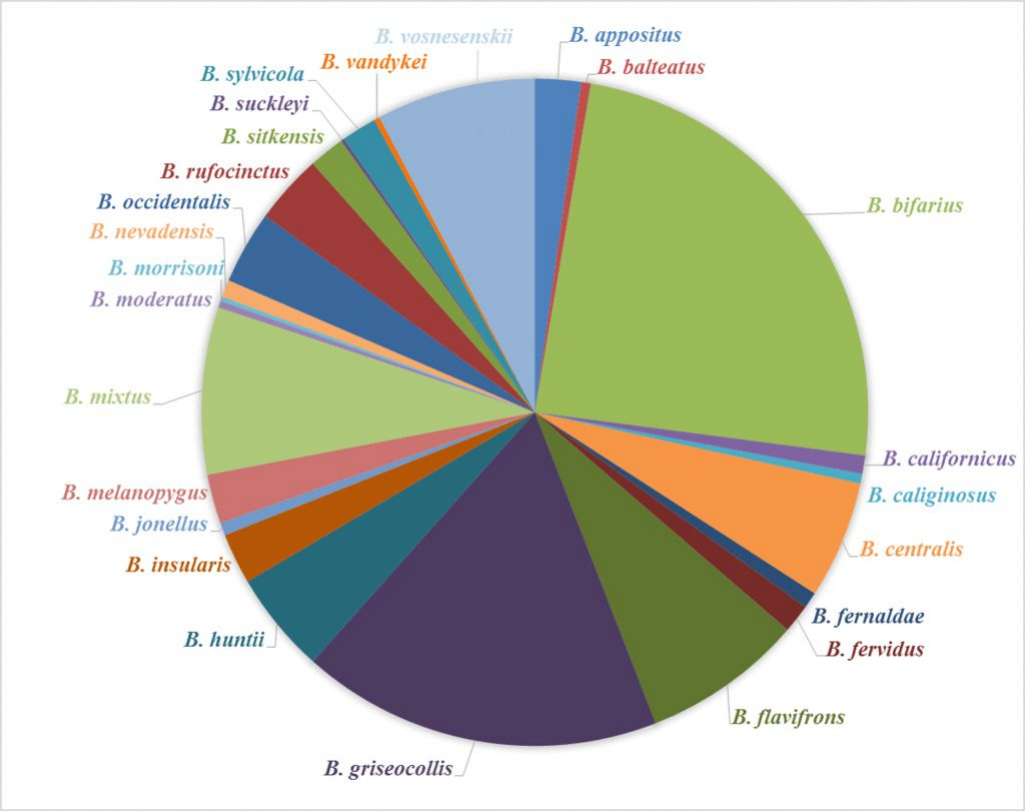
Percentage of specimen records per species detected in the western United States, including Alaska (Suppl. material 1). Western sites are defined as survey sites that are west of the Colorado Rockies (104th western longitude).

**Figure 5.**
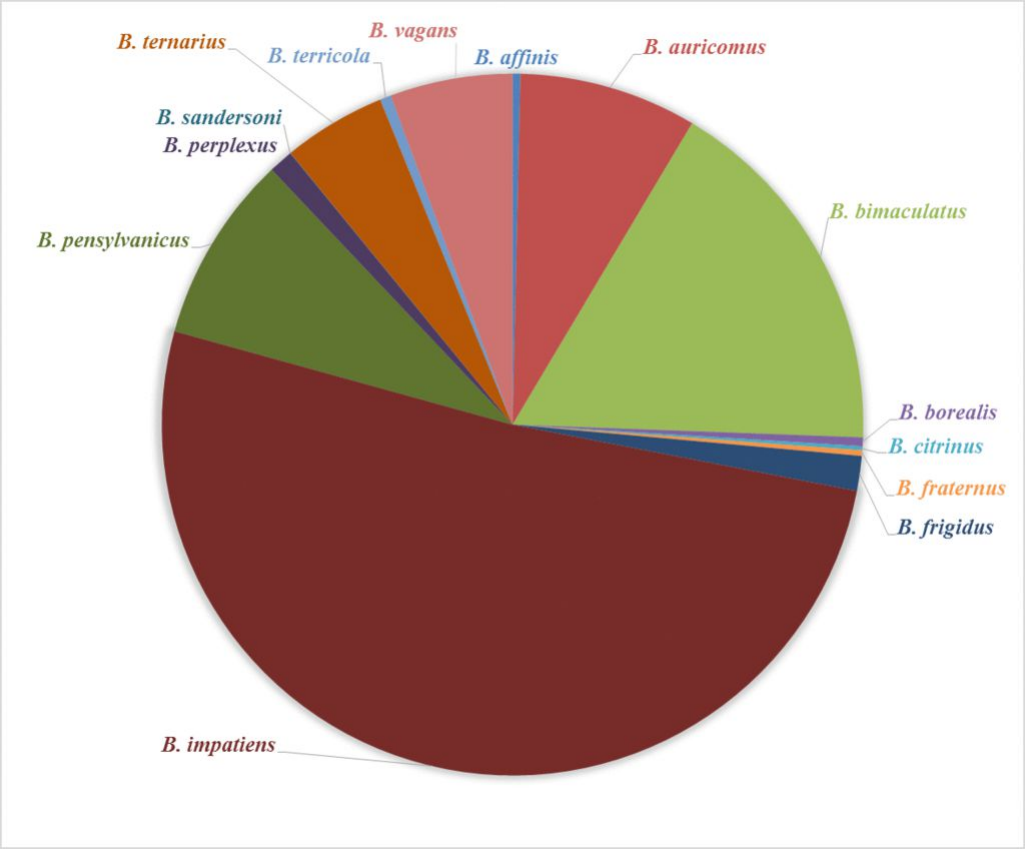
Percentage of specimen records per species detected in the eastern United States. Eastern sites are defined as survey sites that are east of the Colorado Rockies (104th western longitude). Bumble bees that are found in both the western and eastern United States are grouped with the western bumble bee species in Fig. 4 (*e.g.*, *Bombus griseocollis*) (Suppl. material 2).

**Figure 6.**
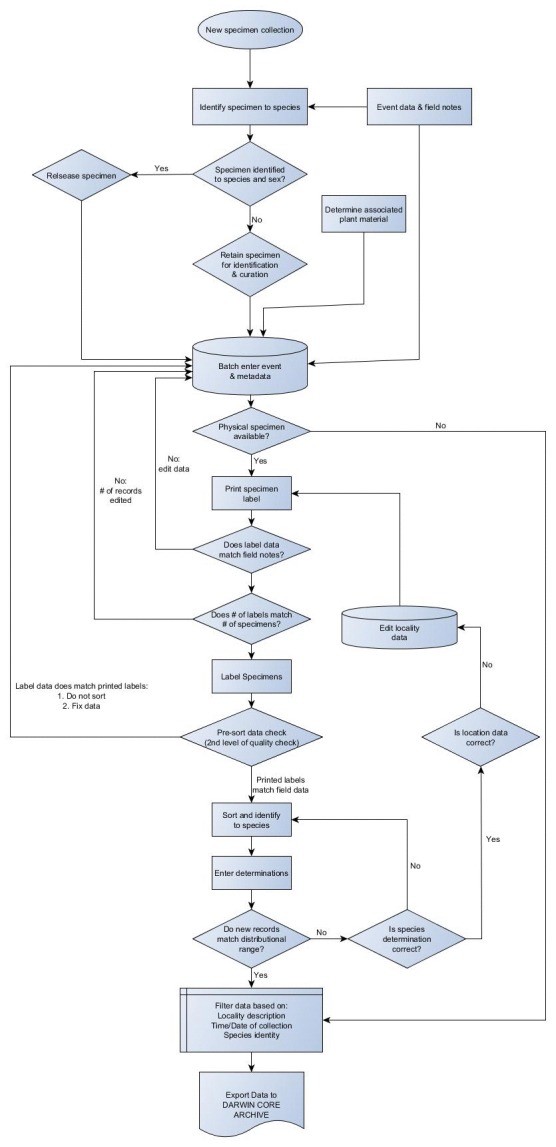
Flowchart for processing of specimen samples at the USDA-ARS Pollinating Insects- Biology, Management, and Systematics Research Laboratory.

 US*Bombus* includes 39 species of the bee genus *Bombus* known to occur in the Nearctic region of the Western Hemisphere (Figs 4, 5). Thus our survey efforts and this dataset document approximately 82% of the described *Bombus* species in North America north of Mexico (Williams et al. 2014). *Bombus* is the only extant genus of the tribe Bombini in the family Apidae. There are an estimated 250 described species across 15 subgenera of *Bombus* worldwide (Williams et al. 2008). Bumble bees are primitively eusocial insects and form colonies in which a division of labor exists among workers (females), drones (male), and queens (females). We differentiate between workers and queens in our dataset with unique identifiers (see dataset description).

In our dataset of North American *Bombus*, the subgenus *Pyrobombus* is the most abundant and most species-rich of the eight subgenera found in the Nearctic. In total, 12,780 bees representing 19 species in the subgenus were detected. In the western United States (including Alaska) the most widespread and abundant bumble bee is *B. bifarius* (Fig. 4), while in the eastern United States the species most abundant is *B. impatiens* (Fig. 5). In addition to being an abundant native bee, *B. impatiens* is commercially reared to pollinate a variety of crops including tomatoes and blueberries (Velthuis and Doorn 2006).

The least abundant and species-poor subgenus detected in our survey was *Alpinobombus*, represented by one species, *B. balteatus*. We also collected four species of bumble bees in the parasitic subgenus *Psithyrus*: *B. insularis*, *B. fernaldae*, *B. suckleyi*, and *B. citrinus*. We did not detect *B. ashtoni* in our survey. *Psithyrus* comprises a unique group of bumble bees in which the females usurp bumble bee colonies, bully or kill the subordinate queen, and use the queen’s daughters to rear her own offspring.

The taxonomic status of three species in our dataset has been debated within the past decade, specifically *B. californicus*, *B. fernaldae*, and *B. moderatus*. Synonymy of *B. californicus* with *B. fervidus* has been proposed by Williams et al. 2014) based on the mitochondrial marker cytochrome oxidase I (COI). Similar taxonomic arguments based on the single gene COI have proposed synonymizing *B. fernaldae* with *B. flavidus* and *B. moderatus* with *B. cryptarum* (Bertsch et al. 2010, Williams et al. 2012). However, these results are at odds with a comprehensive five gene phylogeny of the bumble bees (Cameron et al. 2007), where *B. californicus*, *B. fervidus*, *B. fernaldae*, *B. flavidus*, *B. cryptarum*, and *B. moderatus* were found to be good species. In this dataset we maintain the species status as defined with molecular data by Cameron et al. 2007) and proposed taxonomy by Thorp et al. 1983). Finally, we did not detect *B. cockerelli* (= *B. vagans*) while surveying within its historic range (Fig. 1) (Yanega 2013).

The species with the least number of records in our survey are *B. ashtoni* (n = 0), *B. franklini* (n = 0), *B. sandersoni* (n = 1), *B. citrinus* (n = 11), *B. fraternus* (n = 16), *B. suckleyi* (n = 19), *B. affinis* (n = 22), *B. borealis* (n = 25), *B. terricola* (n = 31), *B. vandykei* (n = 44), and *B. moderatus* (n = 39) (Figs 4, 5). The limited number of *B. terricola* may be due to low survey coverge in the Northeast (Fig. 1) where published species distribution models of *B. terricola* predict to be of high habitat suitability (Cameron et al. 2011). *Bombus franklini*, which was not detected in our survey effort has the smallest known geographic distribution and only occurs in one ecoregion (Koch et al. 2012). At present *B. caliginosus* and *B. morrisoni* are listed as vulnerable by the International Union for the Conservation of Nature (IUCN) while *B. franklini* is listed as critically endangered and *B. fraternus* is listed as endangered (Kevan 2008, Hatfield et al. 2014a, Hatfield et al. 2014b, Hatfield et al. 2014c). However, several other species including *B. affinis* are candidates for listing under the U.S. Endangered Species Act and the IUCN (Jepsen et al. 2013).

All bumble bee species determinations in this dataset have been reviewed by the authors. Specimens not identified to species due to poor physical conditions are included in the dataset as “*Bombus* sp.”.

### Taxa included

**Table:** 

Rank	Scientific Name	Common Name
kingdom	Animalia	
phylum	Arthropoda	
class	Insecta	
order	Hymenoptera	
family	Apidae	
subfamily	Apinae	
tribe	Bombini	
genus	Bombus	bumble bee, bumblebee, humble bee, dumbledore
species	Bombus affinis	Rusty-patched bumble bee
species	Bombus appositus	White-shouldered bumble bee
species	Bombus auricomus	Black and gold bumble bee
species	Bombus balteatus	High country bumble bee
species	Bombus bifarius	Two form bumble bee
species	Bombus bimaculatus	Two-spotted bumble bee
species	Bombus borealis	Northern amber bumble bee
species	Bombus californicus	California bumble bee
species	Bombus caliginosus	Obscure bumble bee
species	Bombus centralis	Central bumble bee
species	Bombus citrinus	Lemon cuckoo bumble bee
species	Bombus fernaldae (=flavidus, in part)	Fernald cuckoo bumble bee
species	Bombus fervidus	Yellow bumble bee
species	Bombus flavifrons	Yellow head bumble bee
species	Bombus fraternus	Southern plains bumble bee
species	Bombus frigidus	Frigid bumble bee
species	Bombus griseocollis	Brown-belted bumble bee
species	Bombus huntii	Hunt bumble bee
species	Bombus impatiens	Common eastern bumble bee
species	Bombus insularis	Indiscriminate cuckoo bumble bee
species	Bombus jonellus	Heath bumble bee
species	Bombus melanopygus	Black tail bumble bee
species	Bombus mixtus	Fuzzy-horned bumble bee
species	Bombus moderatus (=cryptarum, in part)	Cryptic Bumble Bee
species	Bombus morrisoni	Morrison bumble bee
species	Bombus nevadensis	Nevada bumble bee
species	Bombus occidentalis	Western bumble bee
species	Bombus pensylvanicus	American bumble bee
species	Bombus perplexus	Confusing bumble bee
species	Bombus rufocinctus	Red-belted bumble bee
species	Bombus sandersoni	Sanderson bumble bee
species	Bombus sitkensis	Sitka bumble bee
species	Bombus suckleyi	Suckley cuckoo bumble bee
species	Bombus sylvicola	Forest bumble bee
species	Bombus ternarius	Tri-colored bumble bee
species	Bombus terricola	Yellow-banded bumble bee
species	Bombus vagans	Half-black bumble bee
species	Bombus vandykei	van Dyke bumble bee
species	Bombus vosnesenskii	Vosnesensky bumble bee

## Temporal coverage

### Notes

The bumble bee surveys described in US*Bombus* were conducted from 13 July 2007 to 1 August 2010 during the summer months (June - August) when bumble bee female workers in the Northern Hemisphere are actively foraging for nectar and pollen to bring back to their growing colonies. Bumble bees distributed at a low latitude and elevation sites were typically surveyed in early June whereas bumble bees distributed at high latitude and elevation sites were surveyed in late July and early August.

## Collection data

### Collection name

USDA-ARS National Pollinating Insect Collection, Logan, Utah, U.S.A.

### Collection identifier

BBSL & EBOD

### Specimen preservation method

Dried and Pinned Specimens

### Curatorial unit

Of the 17,930 bumble bee records, 9,380 records represent 9,363 dried and pinned adult individuals affixed with label data and matrix barcode. The specimens are housed in standard insect museum drawers and preserved from dermestid beetle damage by routine freezing of drawers at -20°C. All specimens are housed at the U.S. National Pollinating Insect Collection in Logan, Utah and are individually represented by the barcode prefix BBSL. The remaining 831 digital records represent 8,567 bumble bees that were caught and released in the field after identification in the eastern U.S.A. Thus, no pinned specimen or label data are associated with these data. These observation records are represented by the barcode prefix EBOD. All species determinations were made by authorities in bumble bee taxonomy, identification, and natural history.

## Usage rights

### Use license

Creative Commons CCZero

### IP rights notes

This work is licensed under a Creative Commons Attribution-NonCommerical ShareAlike 3.0 Unported Licenses. http://creativecommons.org/licenses/by-nc-sa/3.0/. Records highlighted in the DWC fields “rights” and “rightsholder” indicate specimens have additional usage rights.

## Data resources

### Data package title

USBombus, a database of contemporary survey data for North American Bumble Bees (Hymenoptera, Apidae, *Bombus*) distributed in the United States

### Resource link

http://ipt.pensoft.net/resource?r=usbombus

### Number of data sets

1

### Data set 1.

#### Data set name

USBombus

#### Data format

Darwin Core Archive

#### Number of columns

49

#### Download URL

http://ipt.pensoft.net/resource?r=usbombus

#### Data format version

2.4

#### Description

US*Bombus* is a result of a multidisciplinary study on the conservation status, disease ecology, and genetic diversity of North American bees in the genus *Bombus* in the U.S.A. The database includes 17,930 adult occurrence records across 397 locations and 39 species of *Bombus*. The database is split into two data types. Bees associated with the BBSL prefix represent an individual specimen, whereas bees associated with the EBOD prefix represent a collecting event where the total number of specimens by species and sex are summed. Thus, the total number of Catalog Numbers (*i.e.*, BBSL or EBOD) in US*Bombus* is 10,211. Summation of specimens associated with EBOD are found in the DWC field Individual Count. In total 439 queen, 3,164 male, and 14,327 female (non-queen, *i.e.*, workers) specimens are recorded in this dataset. Each BBSL and EBOD record consist of species name, locality, collector’s name (when available), collection date, time of collection (AM/PM), latitude, longitude, host plants, associated organisms, name of identifier and repository (if applicable). EBOD collectors are represented by the qualifier “University of Illinois and Illinois Natural History Survey”. The Cartesian coordinates for the collection sites were collected with Garmin GPS units in decimal latitude and longitude.

**Data set 1.** 

Column label	Column description
id	Identification Information. OccurrenceID.
type	Pinned Specimen or Observation Record
language	Language (=English)
rights	Rights
rightsHolder	Rights Holder
collectionID	Collection ID
institutionCode	Institution Code
collectionCode	Collection Code
datasetName	Data set Name
ownerInstitutionCode	Owner Institution Code
basisOfRecord	Preserved Specimen or Observation Record
informationWithheld	Information Withheld (Yes, No)
occurrenceID	Occurrence ID
catalogNumber	Catalog Number
recordedBy	Recorded By (i.e., Collectors)
individualCount	Count of Specimens
sex	Female, Female Queen, or Male
otherCatalogNumbers	Other Catalog Numbers
previousIdentifications	Previous Identifications
associatedReferences	Associated References
associatedTaxa	Associated Taxa, e.g., Floral Host
year	Year
month	Month
day	Day
verbatimEventDate	Verbatim Event Date
fieldNumber	Plot ID, if relevant
country	Country
stateProvince	State/Provnce
county	County
locality	Locality Description
verbatimElevation	Verbatim Elevation
minimumElevationInMeters	Elevation based on U.S. DEM (2015)
decimalLatitude	Latitude WGS 1984
decimalLongitude	Longitude WGS 1984
geodeticDatum	Datum (Geospatial)
identifiedBy	Species Identification Author
identificationQualifier	Identification Qualifier
scientificName	Scientific Name
kingdom	Kingdom
phylum	Phylum
class	Class
order	Order
family	Family
genus	Genus
subgenus	Subgenus
specificEpithet	Specific Epithet
infraspecificEpithet	Infraspecific Epithet
taxonRank	Taxon Rank
scientificNameAuthorship	Scientific Name Authorship

## Additional information

**Additional publications based on use of this dataset**

1. Cordes N (2010) The role of pathogens in the decline of North American bumble bees with a focus on the Microsporidium Nosema bombi. MS Thesis. University of Illinois at Urbana-Champaign.
2. Howard, E (2013) Land-use Change and the Decline of the Western Bumble Bee. MS Thesis. The George Washington University.
3. Koch JB (2011) The decline and conservation status of North American bumble bees. MS Thesis, Utah State University.
4. Lozier, J.D., 2014. Revisiting comparisons of genetic diversity in stable and declining species: assessing genome-wide polymorphism in North American bumble bees using RAD sequencing. Molecular Ecology 23, 788–801.
5. Lozier, J.D., Strange, J.P., Koch, J.B., 2013. Landscape heterogeneity predicts gene flow in a widespread polymorphic bumble bee, *Bombus bifarius* (Hymenoptera: Apidae). Conservation Genetics 14, 1099–1110.
6. Lozier, J.D., Strange, J.P., Stewart, I.J., Cameron, S.A., 2011. Patterns of range-wide genetic variation in six North American bumble bee (Apidae: *Bombus*) species. Molecular Ecology 20, 4870–88.
7. Szabo ND, Colla SR, Wagner, DL, Gall, LW, Kerr JT (2012) Do pathogen spillover, pesticide use, or habitat loss explain recent North American bumblebee declines? Conservation Letters 5:232-239.

## Supplementary Material

Supplementary material 1 Count of specimens of each bumble bee species in the western U.S.A. and Alaska Data type: occurences
Brief description: Count of specimens per species in western United States and Alaska, including some species that are found in the Eastern United States.
File: oo_59705.csv Jonathan B. Koch, Jeffrey Lozier, James Strange, Harold Ikerd, Terry Griswold, Nils Cordes, Leellen Solter, Issac Steward, Sydney Cameron

Supplementary material 2 Count of specimens of each bumble bee species in the eastern U.S.A. Data type: occurences
Brief description: Count of specimens per species from in eastern United States. Some species that are also found in the western United States were included in Supplementary Table 1.
File: oo_59706.csv Jonathan B. Koch, Jeffrey Lozier, James Strange, Harold Ikerd, Terry Griswold, Nils Cordes, Leellen Solter, Issac Steward, Sydney Cameron

## Figures and Tables

**Table 1.** Relative abundance of *Bombus* species in the contiguous United States and Alaska by World Wildlife Fund (WWF) ecoregion status (Olson and Dinerstein 2002, Olson et al. 2001). WWF ecoregion status is grouped into three broad categories: critical or endangered, vulnerable, and relatively stable or intact.

**Species**	**# of Ecoregions**	**# of Specimens**	**Ecoregion Status**		
			**Critical or Endangered (%)**	**Vulnerable (%)**	**Relatively Stable** **or Intact (%)**
*B. affinis*	2	22	100	0	0
*B. appositus*	13	260	19	54	27
*B. auricomus*	11	502	86	0	14
*B. balteatus*	5	55	0	36	64
*B. bifarius*	19	2870	35	35	30
*B. bimaculatus*	14	1042	91	9	0
*B. borealis*	7	25	76	20	4
*B. californicus*	10	104	62	17	21
*B. caliginosus*	4	75	95	5	0
*B. centralis*	15	663	20	25	56
*B. citrinus*	3	11	100	0	0
*B. fernaldae*	13	91	41	48	11
*B. fervidus*	19	162	80	7	13
*B. flavifrons*	25	910	40	19	41
*B. fraternus*	5	16	75	25	0
*B. frigidus*	8	98	0	19	81
*B. griseocollis*	29	2042	89	7	4
*B. huntii*	13	577	24	11	65
*B. impatiens*	18	3138	90	9	0
*B. insularis*	18	288	36	30	34
*B. jonellus*	7	72	0	0	100
*B. melanopygus*	18	278	37	39	24
*B. mixtus*	21	945	50	41	9
*B. moderatus*	5	39	0	0	100
*B. morrisoni*	6	25	32	0	68
*B. nevadensis*	11	108	45	26	29
*B. occidentalis*	17	415	6	9	86
*B. pensylvanicus*	16	530	98	1	2
*B. perplexus*	8	69	67	16	17
*B. rufocinctus*	17	395	64	11	25
*B. sandersoni*	1	1	0	100	0
*B. sitkensis*	12	203	38	38	24
*B. suckleyi*	3	19	26	68	5
*B. sylvicola*	13	199	8	31	61
*B. ternarius*	6	291	92	1	6
*B. terricola*	5	31	0	52	48
*B. vagans*	14	346	66	32	3
*B. vandykei*	6	44	78	9	13
*B. vosnesenskii*	8	959	87	13	0
